# Effect of yogurt probiotic liquid and powder supplementation on hematology and biochemistry blood levels of layer-phase laying hens

**DOI:** 10.5455/javar.2024.k843

**Published:** 2024-12-27

**Authors:** Salma Gracela Gurning, Lovita Adriani, Andi Mushawwir, Indrawati Yudha Asmara

**Affiliations:** 1 Department of Animal Nutrition and Feed Technology, Faculty of Animal Sciences, University of Padjadjaran, Sumedang, Indonesia; 2 Department of Livestock Production, Faculty of Animal Sciences, University of Padjadjaran, Sumedang, Indonesia

**Keywords:** Alkaline phosphatase, Cholesterol, Laying-hens, Leucocytes, Probiotics

## Abstract

**Objective:**

The aim of this study was to determine the best form and concentration of yogurt probiotics that can reduce leucocyte, neutrophil, lymphocyte, alkaline phosphatase, and cholesterol levels to normal limits.

**Materials and Methods:**

There were 35 Isa Brown laying hens aged 40 weeks with health conditions chosen as the object of research. We used a completely randomized design method with seven treatments. Treatments included P0: basal feed; T1: basal feed and probiotic liquid 2%; T2: basal feed and probiotic liquid 3%; T3: basal feed and probiotic liquid 4%; T4: basal feed and probiotic powder 2%; T5: basal feed and probiotic powder 3%; and T6: basal feed and probiotic powder 4%. On day 35 of the research, we collected blood samples. We analyzed the data using analysis of variance, followed by Duncan’s multiple range test.

**Results:**

This showed that probiotic liquid and powdered yogurt had a significant effect (*p* < 0.05) on all parameters. Supplementation with 4% probiotic powder reduced neutrophil (53.96%), lymphocyte (27.84%), and N/L ratios (36.25%); alkaline phosphatase levels (53.6%); and cholesterol levels (ApB 26.65% and LDL 42.4%) compared to the control.

**Conclusion:**

This study shows that all probiotic supplementation shows improvement in the studied parameters, but the addition of 4% probiotic powder had the best result for reducing neutrophil, lymphocyte, N/L ratio, alkaline phosphatase, and cholesterol levels compared to the control and probiotic liquid.

## Introduction

Pathogenic bacteria in laying hens’ digestive tracts are triggered by various factors such as poor sanitation and hygiene, contaminated feed and drinking water, and imbalances of microflora in the digestive tract [1]. The immune system will respond to pathogenic bacteria present in the body through several defense mechanisms, including the production of free radicals. Excessively produced free radicals can trigger inflammation, which ultimately results in increased levels of alkaline phosphatase in the blood and triggers organ disease [2].

Pathogenic bacteria that enter the body will be responded to by macrophages as antigen-presenting cells. Macrophages will recognize [3] and bind pathogenic bacteria to activate the inflammatory process through the release of proinflammatory cytokines consisting of IL-1, IL-6, IL-8, and TNF-α [3]. The excessive release of proinflammatory cytokines can damage uncontrolled tissues accompanied by the release of glucocorticoids as feedback [4]. Increased glucocorticoid levels can reduce the number of lymphocytes, increase the number of blood neutrophils, and affect the stress level of livestock.

Inflammatory mechanisms that disrupt neutrophil, lymphocyte, and alkaline phosphatase levels will not occur if the balance of gut microbiota can be maintained within normal limits by adding probiotics to feed. An effective way to provide probiotics in terms of storing and feeding is by using powder form. The probiotic used in this study is a consortium probiotic consisting of *Lactobacillus bulgaricus, Streptococcus thermophilus, Lactobacillus acidophilus*, and *Bifidobacterium bifidum*. They work synergistically to improve health through the improvement of gut microflora. According to previous research, adding 4% probiotic powder with the same bacterial consortium in a chicken’s diet can increase total lymphocytes by 1.54%, decrease the number of neutrophils by 9.87%, and balance the N/L ratio by 35.48%, also leading to increased immunity in laying hens that are 90 weeks old [5]. Another research also proved that the addition of 4% probiotic powder was able to decrease 12.97% cholesterol in broiler chicken [6].

Research conducted by Kumalasari et al. [6] has shown that despite a decrease in the number of lactic acid bacteria, probiotic powder with simple drying techniques provides the same benefits as probiotic liquid. Therefore, research on the addition of probiotic powder with the same method needs to be retested to confirm that probiotic powder from the simple drying method can provide benefits to another phase of laying hens (40 weeks), especially to improve another parameter that has not been tested before. This study also used the addition of probiotic liquid to the feed to compare the best concentration and form of probiotic yogurt that can affect the levels of neutrophils, lymphocytes, alkaline phosphatase, and cholesterol in laying hens.

## Materials and Methods

### Ethical approval

The entire procedure and conduct of this study have been reviewed and confirmed to be acceptable by fulfilling the ethical requirements of animal experimentation by The Ethics Board for Animal Experiments, BATAN, with number: 92/An-RE/2/23.

### Preparation of probiotic liquid and probiotic powder

There are two types of probiotics used in this study, namely probiotic liquid and probiotic powder. Probiotics were made using fresh cow’s milk with the addition of a consortium of bacteria. *Lactobacillus bulgaricus, S. thermophilus, L. acidophilus, *and* B. bifidum,* as much as 5% (v/v), were inoculated into 250 ml of De Man Rogosa and Sharpe medium and incubated at 37°C for 24 h. Probiotic powders were made by adding maltodextrin DE 10–12 (food grade) as much as 5% to the probiotic liquid and then dried using a vacuum oven at 40°C for 48 h.

### Experimental design and feeds

This study was carried out at Padjadjaran University’s Test Farm located in Sumedang, Indonesia, from September to November 2023. Analysis was conducted at the Bioscience Laboratory, Cimahi, Indonesia. Thirty-five 40-week-old Isa Brown strain laying hens with an average weight of 1.51 ± 0.158 kg were randomly assigned to seven treatments and five replicates using a completely randomized design. The treatments given were T0: basal feeds; T1: basal feeds and 2% probiotic liquid; T2: basal feeds and 3% probiotic liquid; T3: basal feeds and 4% probiotic liquid; T4: basal feeds and 2% probiotic powder; T5: basal feeds and 3% probiotic powder; and T6: basal feeds and 4% probiotic powder. The feed used in this study was a commercial complete feed manufactured by PT East Hope Agriculture Indonesia under the brand name EH 711 for laying hens aged 18 weeks and above. The feed was given to livestock as much as 120 gm/head/day and probiotics were added according to the specific treatments based on dosage from previous studies [6–8].

### Blood hematology measurements

Blood samples were collected from 35 laying hens on day 35. Blood samples were taken as much as 3 ml through the external pectoralis vein on the wing vein. Blood samples were stored in 3 ml EDTA tubes containing anticoagulants. Blood samples were used for leukocyte, neutrophil, lymphocyte, alkaline phosphatase, and blood lipid analysis. Leukocyte, neutrophil, and lymphocyte levels were analyzed using a hematology analyzer at the Bioscience Laboratory, Cimahi, Indonesia. Blood alkaline phosphatase enzyme levels were analyzed using Alkaline Phosphatase reagent catalog code 92214 (Biolabo, France) at the Laboratory of Animal Physiology and Biochemistry, Faculty of Animal Science, Padjadjaran University.

### Blood lipid profile analysis

Specific lipid transport proteins apolipoprotein (ApA1, ApA2, ApB, and ApC) were measured with a spectrophotometer following the measurement instructions based on the Randox Kit. Levels of high-density lipoprotein (HDL) and low-density lipoprotein (LDL) were measured with a spectrophotometer by following the measurement instructions based on the Biolabo Kit (Biolabo, France) at the Laboratory of Animal Physiology and Biochemistry, Faculty of Animal Science, Padjadjaran University.

### Statistical analysis

Data were statistically analyzed using analysis of variance to see interactions or significant differences between treatments. Duncan’s multiple range test was conducted as a follow-up test if the results differed significantly (*p* < 0.05). Data were analyzed using IBM SPSS Statistics 25.

## Results

### Quality of pH and bacteria count in yogurt probiotics

Both types of yogurt probiotics in this study were tested for pH and total bacteria contained in them. Probiotic liquid has a pH of 4.20 with a total of 3.82 × 10^7 ^CFU/ml lactic acid bacteria, while probiotic powder has a pH of 4.25 with a total of 8.82 × 10^4^ CFU/ml lactic acid bacteria. Results are shown in Table 1.

### Leukocyte levels

The highest average leukocyte levels were in T0, which was 17.497 × 10^3^ cells/mm^3^, and the lowest in T6, which was 13.984 × 10^3^ cells/mm^3^. In each treatment, there was a decrease in leukocyte levels of 15.9% (T1), 8.33% (T2), 17.25% (T3), 14.39% (T4), 19.09% (T5), and 20.07% (T6). Leukocyte levels were significantly different (*p* < 0.05). Results are shown in Table 2.

### Neutrophil levels

The highest average neutrophil levels were in T0, which was 0.644 × 10^3^ cells/mm^3^, and the lowest in T6, which was 0.296 x 10^3^ cells/mm^3^. In each treatment, there was a decrease in neutrophil levels by 37.33% (T1), 34.09% (T2), 46% (T3), 45.09% (T4), 49.87% (T5), and 53.96% (T6). Neutrophil levels were significantly different (*p* < 0.05). Results are shown in Table 2.

**Table 1.** Quality of pH and bacteria count in yogurt probiotic.

Probiotics Form	pH	Total Lactic Acid Bacteria (CFU/ml)
Probsiotic liquid	4.20	3.82 × 10^7^
Probiotic powder	4.25	8.82 × 10^4^

### Lymphocyte levels

The highest average lymphocyte level was in T0, which was 10,775 × 10^3^ cells/mm^3^, and the lowest in T6, which was 7.775 × 10^3^ cells/mm^3^. In each treatment, there was a decrease in lymphocyte levels of 19.35% (T1), 10.01% (T2), 22.01% (T3), 20.77% (T4), 24.6% (T5), and 27.84% (T6). Lymphocyte levels were significantly different (*p* < 0.05). Results are shown in Table 2.

### Neutrophil-lymphocyte ratio

**Figure 1.**
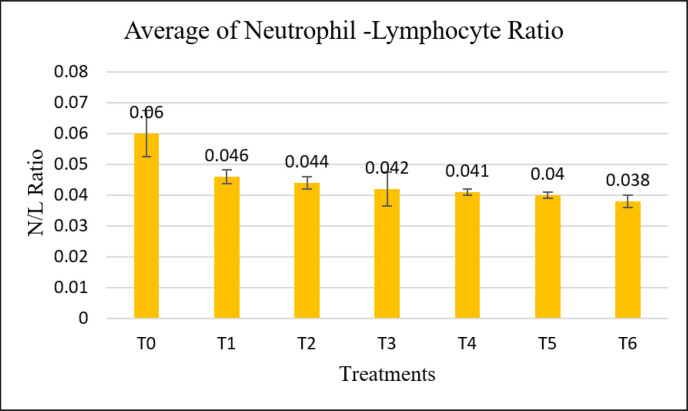
Average of leukocyte, neutrophil, lymphocyte, N/L, and alkaline phosphatase levels.

The highest average ratio N/L was in T0, which was 0.06 dan, and the lowest in T6, which was 0.038. In each treatment, there was a decrease in the ratio N/L of 22.78% (T1), 27.05% (T2), 30.74% (T3), 30.81% (T4), 33.62% (T5), and 36.25% (T6). The ratio N/L was significantly different (*p* < 0.05). The results are described in Figure 1.

### Alkaline phosphatase levels

The highest average ratio was in T0, which was 6.01 U/l, and the lowest in T6, which was 2.79 U/l. In each treatment, there was a decrease in alkaline phosphatase levels of 13.75% (T1), 28.99% (T2), 31.23% (T3), 40.71% (T4), 38.68% (T5), and 53.6% (T6). Alkaline phosphatase levels were significantly different (*p* < 0.05). Results are shown in Table 2.

### Blood lipid profile levels

The result of expression and lipid transport of layer-phase laying hens fed yogurt are shown in Table 3. The result showed that PPAR-γ, ApA1, ApA2, ApC, and HDL had higher (*p* < 0.05) levels in T6 and lower (*p* < 0.05) levels in T0. Meanwhile, ApB and LDL had higher (*p* < 0.05) levels in T0 and lower (*p* < 0.05) levels in T6.

**Table 2.** Average blood hematology levels of laying hens.

Parameters	Treatments						
	T0	T1	T2	T3	T4	T5	T6
Leukocyte (10^3^ cells/mm^3^)	17.497 ± 0.189^a^	14.715 ± 2.02^b^	16.04 ± 0.594^c^	14.478 ± 0.477^b^	14.979 ± 0.167^bc^	14.156 ± 0.129^b^	13.984 ± 0.08^b^
Neutrophils (10^3^ cells/mm^3^)	0.644 ± 0.07^a^	0.404 ± 0.089^bc^	0.424 ± 0.048^b^	0.347 ± 0.047^cd^	0.353 ± 0.012^cd^	0.323 ± 0.00^9^d	0.296 ± 0.011^d^
Lymphocytes (10^3^ cells/mm^3^)	10.775 ± .316^a^	8.69 ± 1.61^b^	9.697 ± 0.675^c^	8.403 ± 0.539^c^	8.537 ± 0.122^c^	8.125 ± 0.023^c^	7.775 ± 0.175^c^
Alkaline Phosphatase (U/l)	6.00 ± 0.077^a^	5.18 ± 0.375^b^	4.27 ± 0.305^c^	4.13 ± 0.25^c^	3.56 ± 0.738^c^	3.68 ± 0.159^c^	2.79 ± 0.202^d^

Notes: The same superscript indicates there is no significant difference (*p* < 0.05). T0 = control diet (basal feeds only), T1: basal feeds and 2% probiotic liquid, T2 = basal feeds and 3% probiotic liquid, T3 = basal feeds and 4% probiotic liquid, T4 = basal feeds and 2% probiotic powder, T5 = basal feeds and 3% probiotic powder, T6 = basal feeds and 4% probiotic powder.

**Table 3.** Regulatory expression and lipid transport of layer-phase laying hens fed yogurt probiotic.

Treatments	Regulator-transpor lipid						
	PPAR- γ (µg/dl)	ApA1 (mg/dl)	ApA2 (mg/dl)	ApB (mg/dl)	ApC (mg/dl)	HDL (mg/dl)	LDL (mg/dl)
T0	4.342^a^	2.608^a^	1.396^ab^	1.381^a^	1.364^a^	1.788^a^	2.816^a^
T1	4.562^a^	3.532^b^	1.392^a^	1.317^a^	1.609^a^	2.442^b^	2.705^a^
T2	4.604^a^	3.621^a^	1.476^c^	1.229^a^	1.882^b^	1.958^c^	2.612^b^
T3	4.692^b^	3.659^c^	2.516^d^	1.075^b^	2.478^c^	2.539^b^	1.748^c^
T4	4.605^a^	3.633^c^	1.651^e^	0.995^c^	1.748^b^	2.428^b^	2.361^d^
T5	4.702^b^	3.642^c^	1.476^c^	1.222^a^	1.779^b^	1.955^c^	2.342^d^
T6	5.173^c^	3.685^d^	2.616^d^	1.013^c^	2.568^d^	2.735^d^	1.622^b^

The different superscript in the same column indicates a significant difference (*p* < 0.05). PPAR-γ:* Peroxisome Proliferator-activated Receptor* γ; ApA1: Apolipoprotein A I; ApA2: Apolipoprotein A-II; ApB: Apolipoprotein B; ApC: Apolipoprotein C; HDL: High Density Lipoprotein; LDL: Low Density Lipoprotein

## Discussion

### Quality of pH and bacteria count in probiotics

The quality of pH and total lactic acid bacteria in probiotic liquid and probiotic powder is shown in Table 1. The decrease in total lactic acid bacteria in probiotic powder can be influenced by the drying process that involves heat because lactic acid bacteria are sensitive to high temperatures. However, even though the drying process of the simple drying technique has an impact on reducing the number of microorganisms, the probiotic powder is still efficacious because the lactic acid bacteria in it can produce a number of beneficial compounds [6], one of which is an antibacterial metabolite compound [7] called bacteriocin. If there are fewer lactic acid bacteria, the bacteriocin activity is also lower.

### Effect of probiotic liquid and probiotic powder concentration on leukocyte levels of laying hens

White blood cells (leukocytes) are one of the blood suspensions that act as the body’s defense system against bacterial, viral, and pathogenic attacks through the mechanism of antibody formation [8]. Observation of leukocyte levels in the blood is used to diagnose livestock health [5]. The number of chicken leukocytes under normal conditions is 12,000–30,000 cells/μl [8]. Based on Table 2, the average leukocyte levels in this study were 13.984 × 10^3^/mm^3^–17.497 × 10^3^/mm^3^. These levels are in the range of normal leukocyte levels.

The high number of leukocytes in control (T0) was due to the absence of the addition of probiotics in the feed. The increase in leukocyte count is influenced by health problems or pathogen attacks [9]. The decrease in the number of leukocytes in chickens treated with liquid and probiotic powder proves that the addition of probiotics to the feed can maintain the balance of microbiota in the digestive tract. The decrease in the number of leukocytes is closely related to the decrease in pathogenic bacteria [10] so if there is no infection of pathogenic bacteria in the body, the number of leukocytes will decrease.

### Effect of probiotic liquid and probiotic powder concentration on neutrophil levels of laying hens

Neutrophils are a type of white blood cell that plays a crucial role in the initial response to infections by phagocytizing pathogenic bacteria [3]. Poultry neutrophil levels in normal conditions range from 4–8 × 10^3^ cells/mm^3^ [11]. Based on Table 2, neutrophil levels in this study were in the range of 0.296 × 10^3^ cells/mm^3^–0.644 × 10^3^ cells/mm^3^. Neutrophil levels in each treatment were below normal limits. Factors that affect neutrophil levels in chickens include genetics, the environment, stress levels, and nutrient adequacy in feed [12].

Higher neutrophil levels in untreated chickens (T0) were due to the absence of the addition of yogurt probiotic containing bacteriocins that are responsible for inhibiting pathogen attacks in the intestine. When pathogens invade the body, they will be recognized and bound by APCs to release proinflammatory cytokines [3]. Proinflammatory cytokines will spread inflammatory signals and direct neutrophils to the site of inflammation so that neutrophils are activated to phagocytize foreign differences, release free radicals, and release new cytokines [13]. The more pathogenic bacteria that enter, the more proinflammatory cytokines are released, so the higher the activation of neutrophils in the blood.

Meanwhile, neutrophil levels decreased in each treatment that was below the normal range, proving that the addition of probiotics can inhibit pathogen attacks to reduce neutrophil production in the blood. The greater decrease in neutrophil levels in chickens treated with probiotic powder can be influenced by the addition of maltodextrin as an encapsulant during the probiotics drying process. Previous research by Tang et al. [2] proved that the addition of probiotics mixed with prebiotics to feed was able to have a greater effect in reducing neutrophil levels by 5.15% and 3.95% compared to the addition of probiotics alone. Therefore, this study reinforces that the addition of probiotic powder yogurt with the highest concentration can reduce neutrophil levels in the blood even though it is below the normal range.

### Effect of probiotic liquid and probiotic powder concentration on lymphocyte levels of laying hens

Lymphocytes are a type of white blood cell that respond to foreign antigens by producing antibodies that circulate in the blood. Lymphocytes are divided into two types, consisting of B lymphocytes that function to attack antigens and T lymphocytes that function to kill antigens and regulate the immune system [11]. The normal range of lymphocyte levels in poultry is 30%–70% [11]. Based on Table 2, the average lymphocyte levels in this study were in the range of 7.775 × 10^3^ cells/mm^3^–10.775 × 10^3^ cells/mm^3 ^(55.6%–61.6%). These levels are at the normal level of lymphocyte levels.

The highest lymphocyte levels in untreated laying hens (T0) were due to the absence of the role of probiotics in attacking pathogens in the body. When the body detects an antigen, B lymphocytes and T lymphocytes in the bone marrow will enter the secondary lymphoid organs to activate the antigen into effector cells and memory cells so that the activated cells will then migrate to the peripheral tissues where the infection occurs [5] so that blood lymphocyte levels increase.

Meanwhile, the decrease in lymphocyte levels in treated chickens was due to decreased pathogen infection due to the work of probiotics. Probiotics play a role in reducing the population of pathogenic bacteria [6] because the lactic acid bacteria contained in them can produce bacteriocins that have bactericidal or bacteriostatic activity [14]. This is in line with previous research, which proves that the decline in pathogen infection also reduces the number of leukocytes and the percentage of lymphocytes [9]. An increase in the number of lymphocytes in the blood is a result of pathogenic microbial infection [15], so a decrease in lymphocyte levels is a response to a decrease in pathogenic infection.

### Effect of probiotic liquid and probiotic powder concentration on neutrophil-lymphocyte ratio of laying hens

Neutrophils and lymphocytes are an important part of the body’s defense system against foreign microorganisms. Of the five types of white blood cells, neutrophils and lymphocytes are the compositions with the largest percentage [3]. The neutrophil-lymphocyte ratio value has three levels, which are low (0.2), normal (0.5), and high (0.8) [5]. The higher the neutrophil-lymphocyte (N/L) ratio, the higher the stress level of the livestock. Based on Table 2, the average N/L ratio in this study was in the range of 0.038–0.06. The N/L ratio in each treatment was below the normal level.

The highest N/L ratio was in untreated laying hens (T0), while the lowest N/L ratio was in laying hens treated with 4% probiotic powder (T6). Previous research on the addition of probiotic powder to the feed of cull-phase laying hens produced N/L ratios in the range of 0.02 to 0.042 [5]. Another study by Tang et al. [2] proved that the addition of probiotics mixed with prebiotics to feed was able to have a greater effect in reducing N/L ratio levels by 3.6% and 9.9% compared to the addition of probiotics alone. Neutrophil-lymphocyte ratios that were below normally indicated that laying hens in each treatment in this study were not stressed. Although the N/L ratio in the study was below the normal range, the addition of probiotic powder yogurt, especially with higher concentrations, proved to be able to reduce the N/L ratio compared to no treatment.

### Effect of probiotic liquid and probiotic powder concentration on alkaline phosphatase levels of laying hens

Alkaline phosphatase (ALP) is a group of isoenzymes in the outer layer of cell membranes that work to catalyze the hydrolysis of organic phosphate esters found in the extracellular space [16]. ALP testing is used to detect liver damage, bile duct blockage, and bone abnormalities. When liver cells are damaged, the liver releases increased alkaline phosphatase into the blood [17], and its activity in serum can increase when there is an injury to the liver due to excessive stress [16].

Based on Table 2, the average alkaline phosphatase levels in this study were in the range of 2.79 U/l–6.00 U/l. The Clinical Diagnostic Division (1990) states that normal ALP levels in chickens are 10–106 U/l [18]. Previous research on hematological and biochemical testing of the blood of male and female Silver Sabahia strain laying hens resulted in serum ALP levels of 295 U/l and 355 U/l [19]. Another study on serum biochemical testing of four 32-week-old crossbreed laying hens reared in a subtropical environment showed serum ALP levels were in the range of 108 U/l to 1100 U/l [10]. Based on some of these research results, it is known that alkaline phosphatase levels in poultry are quite variable depending on the strain, age, sex, treatment, feed, environment, bone, liver, and tissue conditions [20,21,22], also in ruminants [23]. Therefore, until now there is no alkaline phosphatase standard that can be used as a reference in research. Another alternative to prove the effect of treatment on serum alkaline phosphatase levels is to compare the effect of treatment with control.

In this study, the highest average alkaline phosphatase level was in untreated laying hens (T0), while the lowest average alkaline phosphatase level was in laying hens treated with 4% probiotic powder (T6). Another enzyme that is commonly found in the liver and becomes a more specific biomarker of liver function is SGPT (serum glutamate pyruvate transaminase) [24]. Previous research on the effect of probiotic powder on liver function in late-phase laying hens stated that the addition of 4% probiotic powder resulted in SGPT levels of 17.63 IU/l, which decreased by 29.38% when compared to the control [24]. This supports the research that the decrease in ALP in treated chickens shows that adding probiotic liquid yogurt and powder with greater concentrations can improve liver function. In addition to liver function, a decrease in ALP also enhances the function of other organs, namely bones and bile ducts [25].

### Effect of probiotic liquid and probiotic powder concentration on regulatory expression and lipid transport of laying hens

Peroxisome proliferator-activated receptor is a part of the superfamily of ligand-dependent transcription factors that can regulate immunity and inflammation [26] and also regulate metabolic processes such as lipid and glucose homeostasis [17]. This study showed that treatment T1 until T6 significantly increases PPAR-γ (*p* < 0.05). Higher expression of peroxisome proliferator-activated receptors indicates that it regulates lipid metabolism [27] is involved in the regulation of adipogenesis, energy balance, and lipid biosynthesis [25], so that changes in PPAR-γ concentrations are related to changes in the profiles of Apo-A1 and Apo-A2, Apo-C with HDL, and also Apo-B with LDL.

Table 3 shows that yogurt probiotics can increase (*p *< 0.05) Apolipoprotein A I (Apo-A1), Apolipoprotein A-II (Apo-A2), Apolipoprotein C (Apo-C), and HDL levels and also decrease (*p* < 0.05) Apolipoprotein (Apo-B) and LDL levels. Apolipoproteins are the protein component of plasma lipoproteins that bind and transport blood lipids to various body tissues for metabolism. The apolipoproteins are mainly synthesized in the liver [28]. Apolipoprotein A-I (ApA1), Apolipoprotein A-2 (ApA2), and Apolipoprotein C are the components of HDL; meanwhile, ApB is the component of LDL [29]. The levels of apolipoprotein, HDL, and LDL in this study are based on the effect of yogurt probiotics.

Probiotics can produce short-chain fatty acids that contain propionic, acetate, and butyrate acids. Propionic acid can decrease levels of cholesterol by inhibiting HMG-CoA reductase enzyme activity, which is related to cholesterol biosynthesis [6]. Butyrate acid can inhibit cholesterol synthesis in the liver. The cholesterol decrease is also affected by bile salt hydrolase (BSH) production. Lactic acid bacteria in yogurt probiotics can produce BSH that can deconjugate bile acids as free cholic acid. Bile acids that are deconjugated will be excreted in the feces, so the number of bile acids that return to the liver will decrease. Then to balance the amount of bile acids, the liver will take cholesterol in the blood as a precursor to synthesize bile salts. This process will reduce cholesterol levels in the whole blood in the chicken’s body [30].

According to a previous study, dried probiotics with the same bacteria can reduce blood cholesterol levels in broiler chickens [6]. Another research also concludes that adding 4% probiotic treatment can decrease blood cholesterol levels compared to control [6]. A previous study about the effect of supplementation with probiotic liquid and powder in laying hens has resulted in the finding that adding 4% yogurt probiotic gives an optimum result in reducing cholesterol levels [30].

## Conclusion

The addition of liquid and probiotic powder to the feed of 40-week-old laying hens had a significant effect. The results showed a significant improvement in all parameters upon the addition of probiotic liquid and powder. This study demonstrated that the addition of 4% yogurt probiotic powder significantly reduced neutrophil, lymphocyte, and N/L ratios; alkaline phosphatase levels; and cholesterol levels when compared to the control and probiotic liquid.

## List of abbreviations

ALP, alkaline phosphatase; ApA1, apolipoprotein A1; ApA2, apolipoprotein A2; ApB, apolipoprotein B; ApC, apolipoprotein C; APCs, antigen-presenting cells; CFU, colony-forming unit; dl, deciliter; IL, interleukin; HDL, high-density lipoprotein; LDL, low-density lipoprotein; pH, potential of hydrogen; mg, milligrams; mm, millimeters; PPAR- γ, Peroxisome Proliferator-Activated Receptors- γ; SGPT, Serum Glutamate Pyruvate Transaminase; TNF-α, tumor necrosis factor-α; U/l, units per Litre; µg, microgram.

## References

[1] Kusmayadi A, Rahayu N (2020). Total bakteri asam laktat dan coliform usus itik cihateup yang diberi pakan mengandung kombinasi tepung kulit manggis dan kunyit. JITP.

[2] Tang SGH, Sieo CC, Ramasamy K, Wan Zuhainis Saad WZ, Wong HK, Ho YW (2017). Performance, biochemical and haematological responses, and relative organ weights of laying hens fed diets supplemented with prebiotic, probiotic and synbiotic. BMC Vet Res.

[3] Hanifarizani RD, Santoso S, Indrawan IWA (2018). Pengaruh ekstrak etanol daun turi merah terhadap jumlah koloni bakteri di hepar dan kadar TGF-β mencit nifas yang diinokulasi *Staphylococcus aureus*. IJM.

[4] Citrashanty I, Suyoso S, Rahmadewi (2014). Insufisiensi adrenal sekunder pada eritema nodosum leprosum: studi profil TNF-α dan kortisol serum. Berk Ilmu Kesehat.

[5] Adriani L, Kumalasari C, Rohandi, Joni M, Latipudin D (2022). Effect of probiotic powder on the leukocyte, heterophil and lymphocyte level on laying hens. Anim Sci.

[6] Kumalasari C, Muchtaridi, Setiawan I, Adriani L (2020). The application of probiotic drying with simple methods and effect on blood cholesterol levels chicken broiler. Rasayyan J Chem.

[7] Indira M, Venkateswarulu TC, Peele KA, Bobby MDN, Krupanidhi S (2019). Bioactive molecules of probiotic bacteria and their mechanism of action: a review. Biotech.

[8] Jannah PN, Sugiharto S, Isroli I (2017). Jumlah leukosit dan diferensiasi leukosit ayam broiler yang diberi minum air rebusan kunyit. Trop J Anim Sci.

[9] Djaelani MA, Kasiyati K, Sunarno S (2020). Jumlah leukosit, persentase limfosit dan persentase monosit ayam petelur jantan setelah perlakuan penambahan serbuk daun kelor pada pakan. Trop Plant Biol.

[10] Adriani L, Latipudin D, Joni IM, Panatarani C, Sania G (2021). Hematological status and egg production of laying hen with probiotic powder as feed supplements. Earth Environ Sci.

[11] Soeharsono, Adriani L, Hernawan E, Kamil KA, Mushawwir A (2010). Livestock physiology.

[12] Purnomo D, Sugiharto S, Isroli I (2016). Total leukosit dan diferensial leukosit darah ayam broiler akibat penggunaan tepung onggok fermentasi rhizopus oryzae pada ransum. JIIIP.

[13] Tangkas M (2023). Artikel review: sistem imunitas dalam kehamilan. Arc Com Health.

[14] Jozefiak D, Sip A (2013). Bacteriocins in poultry nutrition—a review. Ann Anim Sci.

[15] Mushawwir A (2010). Studi terhadap profil hematologik dan albumin darah ayam ras petelur selama force molting. J Sain Peternak Indones.

[16] Lowe D, Sanvictores T, Zubair M, John S (2023). Alkaline phosphatase.

[17] Hasan KMM, Tamanna N, Haque MA (2018). Biochemical and histopathological profiling of wistar rat treated with brassica napus as a supplementary feed. Food Sci Human Well.

[18] Khawaja T, Khan SH, Mukhtar N, Parveen A, Fareed G (2013). Production performance, egg quality, and biochemical parameters of three way crossbred chickens in sub-tropical environment. Ital J Anim Sci.

[19] El-Sahn AA, Aly OM, El-Turky AI, Afiffi YK, Balat MM, Mosaad N (2022). Hematology and biochemistry profile of silver sabahia chicken strain. Egypt J Biol Pest Control.

[20] Osadcha YV, Sakhatsky MI, Kulibaba RO (2021). Serum clinical biochemical markers of hy-line W-36 laying hens under the influence of increased stocking densities in cages of multilevel batteries. Regul Mech Biosyst.

[21] Mushawwir A, Permana R, Darwis D, Puspitasari T, Pangerteni DS, Nuryanthi N (2021a). Enhancement of the liver histologic of broiler induced by irradiated chitosan (IC). AIP Conf Proc.

[22] Mushawwir A, Permana R, Latipudin D, Suwarno N (2021b). Organic Diallyl-n-Sulfide (Dn-S) inhibited the glycogenolysis pathway and heart failure of heat-stressed laying hens. Earth Environ Sci.

[23] Tanuwiria UH, Susilawati I, Tasripin DS, Salman LB, Mushawwir A (2022). Behavioral, physiological, and blood biochemistry of Friesian Holstein dairy cattle at different altitudes in West Java, Indonesia. Biodiversitas.

[24] Adriani L, Darmadi A, Latipudin D, Rusmana D, Widjastuti T (2022). Improving the level of serum glutamate oxaloacetate transaminase (SGOT) and serum glutamate piruvate transaminase (SGPT) due to the addition of probiotic powder in phase layer chicken. Anim Food Sci J.

[25] Grygiel-Górniak B (2014). Peroxisome proliferator-activated receptors and their ligands: nutritional and clinical implications - a review. Nutr J.

[26] He K, Li Y, Yang K, Gong JP, Li PZ (2014). Effect of peroxisome proliferator-activated receptor γ on the cholesterol efflux of peritoneal macrophages in inflammation. Mol Med Rep.

[27] Muzio G, Barrera G, Pizzimenti S (2021). Peroxisome proliferator-activated receptors (PPARs) and oxidative stress in physiological conditions and in cancer. Antioxidants.

[28] Liu T, Chen JM, Zhang D, Zhang Q, Peng B, Xu L (2021). ApoPred: identification of apolipoproteins and their subfamilies with multifarious features. Front Cell Dev Biol.

[29] Subramanian SP, dan Gundry RL (2022). The known unknows of apolipoprotein glycoylation in health and disease. iScience.

[30] Firmansyah A, Adriani L, Mushawwir A, Mayasari N, Rusmana D, Ishmayana A (2024). Effect of feed supplementation with liquid and powdered yogurt probiotic on the lipid profile of chicken egg yolk. Adv Anim Vet Sci.

